# Guidelines for outpatient administration of naxitamab: Experience from Atrium Health Levine Children's Hospital

**DOI:** 10.1002/cam4.7045

**Published:** 2024-02-23

**Authors:** Erin Murphy Trovillion, Meghan Michael, Cathryn C. Jordan, Lauren Brown, Katlin Phillips, Javier Oesterheld, Giselle Saulnier‐Sholler

**Affiliations:** ^1^ Atrium Health Levine Children's Hospital Charlotte North Carolina USA

**Keywords:** immunotherapy, naxitamab, neuroblastoma

## Abstract

**Aim:**

In this publication, we will share our experience of AE management, provide guidance for appropriate staffing, and the discuss the importance of patient education when treating patients with R/R HR neuroblastoma using naxitamab.

**Background:**

Approved treatments for patients with refractory and/or relapsed (R/R) high‐risk (HR) neuroblastoma are limited, and there is a high unmet need for new treatment combinations. Naxitamab is a disialoganglioside 2 (GD2)‐binding antibody that was approved by the United States Food and Drug Administration in 2020 for use in combination with granulocyte–macrophage colony‐stimulating factor for the treatment of patients with R/R HR neuroblastoma in the bone and/or bone marrow and who have demonstrated a partial response, minor response, or stable disease with prior therapy.

**Methods:**

The pediatric oncology team at Atrium Health Levine Children’s Hospital has successfully treated several patients with naxitamab both alone and in combination with chemotherapy, with no patients requiring unplanned overnight hospitalization and few severe adverse events (AEs). To accomplish this, the team at Levine Children’s Hospital established standard operating procedures for naxitamab, a therapy defined as high acuity due to the potential for acute AEs with rapid onset and that benefits from continuous monitoring by a nursing team and a dedicated provider.

**Conclusions:**

This will provide a practical guide for institutions offering naxitamab to their patients, and ensure successful administration of this high acuity treatment in the outpatient setting.

## 1 INTRODUCTION

Approximately 50% of patients with neuroblastoma are categorized as having high‐risk (HR) disease, and despite important advances in treatment, HR neuroblastoma remains challenging to treat.^1^, ^2^ Approximately 70% of patients with HR neuroblastoma have metastatic disease at diagnosis,^3^ and metastases in the bone and/or bone marrow are associated with high mortality rates.^4^ Bone and bone marrow may act as a reservoir for residual disease, harboring drug‐resistant neuroblastoma cells that may drive refractory disease, and relapse.^5^, ^6^

In recent years, immunotherapy with anti‐disialoganglioside 2 (anti‐GD2) monoclonal antibodies (mAbs; e.g., dinutuximab and naxitamab) has become available for patients with HR neuroblastoma. However, optimization of the treatment regimen is still needed to improve patient outcomes.^7^, ^8^, ^9^

The team at Atrium Health Levine Children's Hospital first started using naxitamab in February of 2021 and rapidly became recognized as a center for excellence with colleagues from across the United States requesting their guidance on how to establish a team to administer naxitamab safely and successfully.

This publication describes how the Levine Children's Hospital team introduced naxitamab into their neuroblastoma treatment program, providing a practical guide for other physicians looking to offer naxitamab therapy to their patients.

### 1.1 Naxitamab for refractory and/or relapsed neuroblastoma

Naxitamab is a humanized GD2‐binding mAb that was granted accelerated United States Food and Drug Administration (FDA) approval in 2020 for use in combination with granulocyte–macrophage colony‐stimulating factor (GM‐CSF). It is indicated for the treatment of pediatric patients 1 year of age and older and adult patients with refractory and/or relapsed (R/R) HR neuroblastoma in the bone and/or bone marrow and who have demonstrated a partial response, minor response, or stable disease with prior therapy.

Compared with other anti‐GD2 mAbs, naxitamab has a short infusion time and can be administered without overnight hospitalization, making it an attractive option for both patients, who often prefer to remain outpatient for their treatment, and their caregivers.^10^ On Cycle 1, Day 1, a 60‐min infusion is recommended, with subsequent infusions (Cycle 1, Days 3, and Day 5, and all other cycles) administered over 30 min, if the initial infusion was well tolerated. The dose of each infusion is 3 mg/kg/day to a maximum of 150 mg/day.

## 2 INTRODUCING NAXITAMAB INTO THE CLINIC

### 2.1 The Atrium Health Levine Children's Hospital experience and team

**FIGURE 1:**
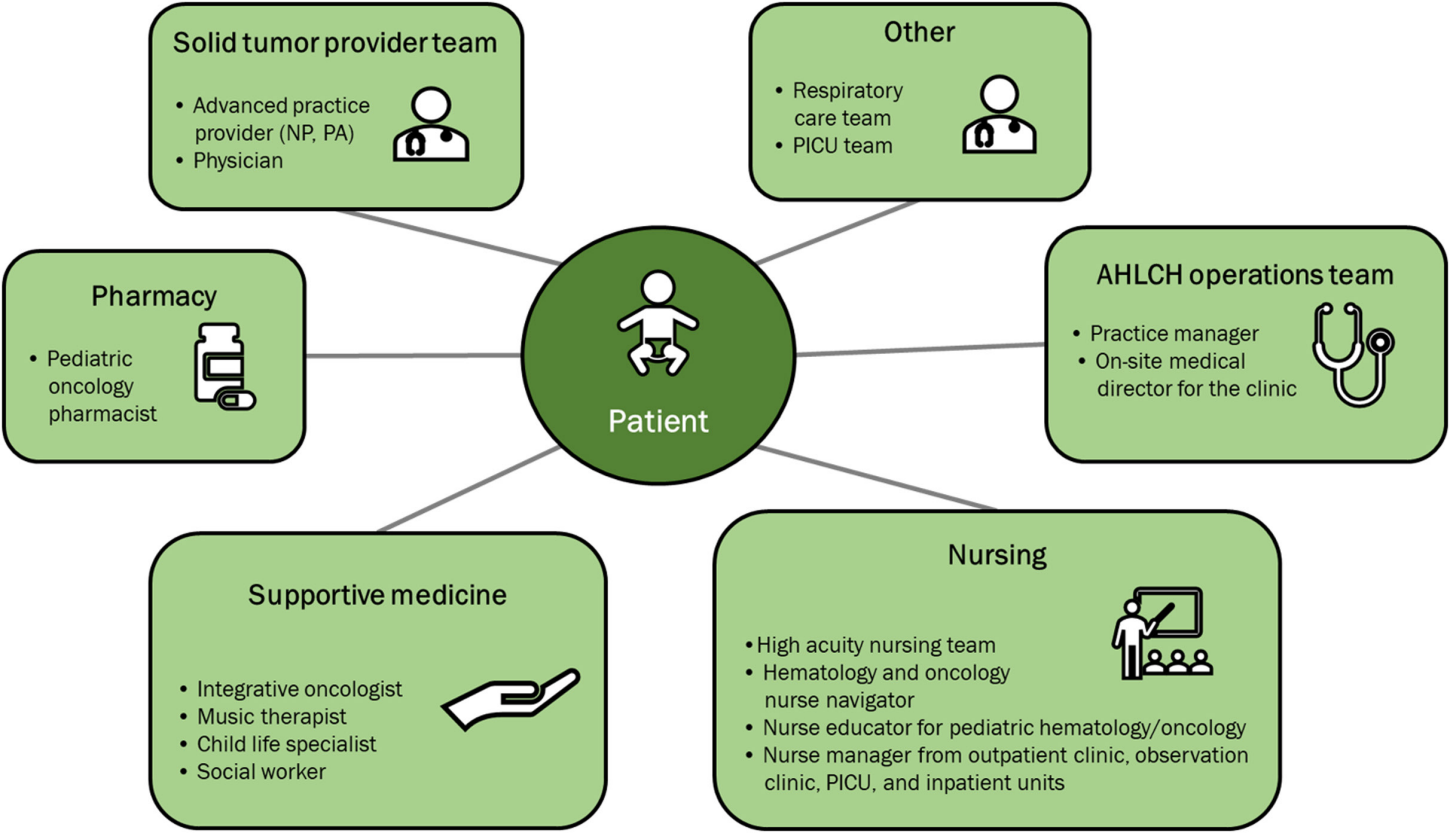
Overview of the Multidisciplinary Team at Atrium Health Levine Children's Hospital. NP, nurse practitioner; PA, physician assistant; PICU, pediatric intensive care unit.

A multidisciplinary team was assembled to manage naxitamab infusions, with key stakeholders including the high acuity nursing team, the solid tumor provider team (physicians [MDs] and advanced practice providers [APPs]), the pediatric oncology pharmacist, and the nurse educator for pediatric hematology and oncology (Figure 1).

While most of our initial referrals were patients at our home institution before initiating naxitamab, we now have new patients who are coming to us specifically to be treated with naxitamab. This has created a need for virtual medical‐care planning calls with the patient and their family before they arrive for their first clinical appointment. These calls are held with our providers and RN navigator, who give an overview of treatment, including what to expect, and carry out an assessment to determine if the patient is a suitable candidate for naxitamab therapy.

A high acuity nursing team comprising a group of pediatric infusion nurses who are highly experienced with infusion‐based therapies, particularly those with the potential for acute or rapidly developing adverse events (AEs) that benefit from constant monitoring, is utilized for each infusion. Initial infusions were administered in our observation unit, with additional nursing support provided by a hematology/oncology nurse. The pediatric intensive care unit (PICU) rapid‐response team was made aware of these planned infusions and were notified when the infusions began. After six infusions were successfully completed in the observation unit, the infusions were moved to the outpatient pediatric hematology/oncology clinic, where they are administered by the same experienced nursing team.

Following each naxitamab infusion, all key stakeholders attend a short debrief focusing on the current patient, how the infusion went, and what could have been done to improve the infusion experience for the patient, caregiver, and team; what AEs were experienced and how they were managed; sharing of new or updated standard operating procedures (SOPs); and discussion of any group tasks. Team debriefing after infusions is valuable, particularly when first introducing naxitamab into the neuroblastoma treatment armamentarium, following the initial infusion, or if AEs are reported that had not been seen in that patient before.

Training of nurses caring for inpatients with neuroblastoma is now taking place at the Levine Children's Hospital to facilitate naxitamab administration as part of frontline neuroblastoma treatment through a Beat Childhood Cancer (BCC) clinical trial, BCC018 (NCT05489887).

### 2.2 Setting up and administering naxitamab

Bringing a high acuity therapy like naxitamab to our institution highlighted the need to create a process that would allow us to ensure its safe and effective use in clinical practice. Several documents were specially developed by the Levine Children's Hospital team to ensure all relevant information was readily available to those who need it.

A “Naxitamab Roles and Responsibilities” SOP was developed, setting out team duties including initial family contact, and scheduling (RN navigator); establishing the required administrative accounts and ensuring team members have appropriate access to patient medical records (referrals coordinator/authorization specialist); financial and travel assistance for the patient and their caregivers (global health services); ensuring availability of medication and obtaining any necessary approvals (pharmacy); review of patient records and ensuring appropriate medication orders and infusion staffing (providers).

In the Appendix S1 shows the roles and responsibilities for naxitamab treatment at Levine Children's Hospital, Appendix S2 the new therapy submission form, and Appendix S3 the new project timeline.

The pharmacy department was instrumental in bringing naxitamab into our clinical practice, and outlined the steps required to safely administer naxitamab at Levine Children's Hospital. A Sterile Compounding Record for naxitamab, acquisition of naxitamab via manufacturer‐specified purchasing programs, and development of naxitamab premedication regimens (including dose, route, and frequency of administration) and supportive care plans were implemented. The pharmacy team also ensured availability of premedication and supportive therapies at patient‐specific doses; alternatively, an automated medication dispensing cabinet could be used, allowing easy access to required medications from the treatment room. As key team members, pharmacists participated in multidisciplinary care‐planning meetings where patient‐specific supportive care plans were developed based on previous intolerance or allergy to therapies.

To consolidate treatment information and provide a useful reference for the team, an acute supportive‐care plan (Appendix S4) that includes standardized order sets was created and is now utilized in the electronic medical record (Epic Beacon treatment plans by Surety Systems, Raleigh, NC, USA). This captures key patient information including weight, height, allergies, and whether they are receiving naxitamab as part of a study. The various premedications that should be administered are detailed, starting with basic fluid needs (intravenous [i.v.] 0.9% normal saline or options such as 5% dextrose and 0.45% normal saline), before explaining how to adjust doses of naxitamab or GM‐CSF to account for patient weight. Premedications are listed according to when they should be administered (e.g., 60 min [cetirizine, methylprednisolone, and oxycodone] or 30 min [diphenhydramine, famotidine, and acetaminophen] before each naxitamab dose). Throughout there are helpful reminders to the team (e.g., for GM‐CSF, it must be administered at least 1 h before naxitamab, and on Days 2 and 4, the patient/caregiver will be responsible for administration; for naxitamab, while the initial infusion should take place over 60 min, subsequent doses may be infused over 30 min, if tolerated). A useful checklist is provided for nurses, highlighting the need for three points of i.v. access, constant cardiac rhythm and O_2_ saturation monitoring starting before the infusion, vital sign checks (pre‐infusion, every 5 min during infusion, and between infusion and discharge [2 × 15 min, 1 × 30 min post‐infusion]), and that hot/cold packs should be available at bedside. The care plan also details AE management, as discussed in the AE management section.

On infusion days, a supply cart is positioned outside the patient's room. This can be quickly brought into the room as needed, and includes needles, syringes, i.v. tubing, and respiratory items (e.g., nasal cannula, simple face mask, non‐rebreather mask, inhaled medication administration equipment) all prepared for immediate use. In the patient's room, a bedside commode can also be useful.

To safeguard staffing levels and provide a schedule for drug administration, a “high acuity calendar” was developed for review at weekly multidisciplinary team meetings. A range of patient education materials are available, many developed in collaboration with child life specialists, and including a presentation for the family that details what to expect before, during, and after an infusion, and a blood pressure log for use by caregivers during home monitoring.

We also enrolled in Y‐mAbs Connect® (Y‐mAbs Therapeutics, Inc., New York, NY, USA), a patient support program that provides information about access, insurance, financial support, and a range of other resources for both health‐care providers and patients and their caregivers. Many of our patient and caregiver materials were developed using this resource.

### 2.3 Adverse event management

The first infusion of naxitamab is often the most challenging for both the patient and the infusion team. Every patient is unique, but pain and hypotension are almost universally experienced. After the initial infusion, the team is aware of how the patient responds to naxitamab, and subsequent infusions can be tailored to individual patient needs. To date, our team has treated more than 10 patients with naxitamab and has administered more than 150 doses. In Table 1, we provide a few patient examples of AEs that were experienced during the naxitamab infusions and how it was managed by the team. All AEs were successfully managed by the team without needing hospital admission.

**TABLE 1:** Patient examples of AEs experienced, and treatment/management provided by infusion team.

Patient Number	Sex	# of Naxitamab Doses Received	First Time Receiving Naxitamab	AE	Grade (CTCAE v. 4)	# of Instances	Treatment/Management
1	M	6	No	Pain Hypotension Hives	2 2 2	6 1 1	Dilaudid given as needed with improvement of pain. NSB, paused infusion; resolution and infusion completed. Benadryl and hydroxyzine given; resolution.
2	F	3	Yes	Pain Emesis	2 2	2 1	Dilaudid given as needed with improvement of pain. Zofran given with improvement.
3	F	15	Yes	Pain Hypertension Hypotension	2 3 2	15 8 5	Dilaudid given as needed with improvement of pain. Labetalol given with improvement; no admission required. NSB; infusion continued; resolution and infusion completed.
4	M	6	Yes	Pain Hypotension Angioedema	2 2 2	2 1 4	Dilaudid given as needed with improvement of pain. NSB; infusion continued; resolution and infusion completed. No intervention during infusion; Solumedrol given as premedication for all subsequent infusions.

On the first day of naxitamab infusion, all emergency medications (including epinephrine) are drawn up and ready at the patient's bedside for immediate use, as needed, during the infusion. For subsequent infusions, pain medications and a normal saline bolus are prepared, with other emergency medicines available at bedside but not prepared.

The prescribing information^11^ and a recent publication from Mora et al.^7^ provide a thorough overview of the recommended premedication and supportive therapy strategies to follow when using naxitamab. These include when to use each medication and at what dose, in addition to providing algorithms that detail when to pause the naxitamab infusion, when to reinitiate and at what rate, and when to consider permanent discontinuation of naxitamab. To supplement these materials, the team at Levine Children's hospital have developed guidance based on what they have found helpful in their experience. This is outlined below and in Appendix S4 (Protocol: Naxitamab for R/R HR neuroblastoma at Levine Children's Hospital) and Appendix S5 (Nurse Checklist for Naxitamab Treatment at Levine Children's Hospital).

The care plan (Appendix S4) details AE management for infusion‐related reactions (IRRs), hypertension, neurotoxicity, and other AEs. IRRs are defined as a range of AEs that occur in response to the infusion: these include hypersensitivity reactions such as urticaria and anaphylaxis, as well as respiratory symptoms and hypotension. Supportive treatments for IRRs, defined as those that should be available at bedside during the infusion, include 1 mg/mL intramuscular (i.m.) epinephrine every 5–15 min (0.01 mg/kg/dose, maximum dose 0.5 mg) for respiratory distress, airway obstruction, or angioedema, with nebulized racepinephrine suggested at a lower frequency for stridor, bronchospasm, or upper airway obstruction; nebulized albuterol is also advised for wheezing. For hypotension, i.v. 0.9% normal saline bolus is delivered via push/pull administration utilizing a three‐way stopcock method.

It is important to note that naxitamab should not be initiated in patients with uncontrolled hypertension; i.v. labetalol (0.2–1 mg/kg/dose, maximum dose 40 mg) over 2 min (maximum dose 10 mg/min) is recommended for patients who develop hypertension during an infusion of naxitamab. Hypertension is defined as systolic and/or diastolic blood pressure greater than 5 mmHg above the 99th percentile for age and height.

For severe pain (based on patient report or signs such as elevated heart rate or facial grimacing), i.v. hydromorphone (0.00375–0.015 mg/kg/dose, maximum dose 0.2 mg) or morphine (0.025–0.1 mg/kg/dose, maximum dose 1 mg) is recommended over 1–5 min every 5 min to a maximum of four doses, with use beyond this at provider discretion. The choice between morphine and hydromorphone is based on patient and caregiver preference. A single i.v. dose of ketorolac (0.5 mg/kg/dose, maximum dose 30 mg) is suggested for moderate pain and, if utilized, is often given before subsequent infusions as a premedication, 30 min before the infusion. Ideally, pain medications should be administered as soon as pain is experienced, rather than waiting for severe symptoms to develop. If respiratory depression occurs due to opioid overuse (e.g., a respiratory rate of ≤12 breaths/min and/or patient is receiving oxygen but unable to maintain oxygen saturation of ≥90%), i.v. naloxone (0.001 mg/kg/dose) should be administered every 3 min. A single dose of i.v. lorazepam (0.01–0.02 mg/kg/dose, maximum dose 1 mg) is recommended for anxiety during the infusion. Supplemental oxygen should always be available.

As part of the integrated approach used at Levine Children's Hospital, the child life specialist plays a pivotal role in patient education (“what to expect”) and assists in developing a plan for during the infusion. This plan is tailored to the patient's preferences and can involve resources for the caregivers or one‐on‐one activities with the patient. Some patients, for example, may enjoy making window art and being fully distracted, requiring minimal supportive medication, whereas others may request a countdown timer so they know when the infusion will complete.^7^, ^9^, ^12^

Music therapy can be used before naxitamab infusion to decrease anxiety and normalize the environment via distraction. This can take the form of either music‐making or listening to music, tailored to the patients' preferences. If the patient responds well to music therapy during premedication, these activities can continue into the infusion. However, for some patients, music‐assisted relaxation is preferred; this can involve imagery or progressive muscle relaxation. Application of the Iso Principle can also be effective, where the pace of the preferred music is first matched to the patients breathing rate, and then slowed to facilitate deeper breathing.^13^

## 3 PRACTICAL CONSIDERATIONS

Our experience has shown that a prepared team equals a calm team that is ready to act and respond quickly. When working with a multidisciplinary team, it is important to ensure all are invested in bringing new therapies to the institution, and that there is open communication and agreement from all key stakeholders. Furthermore, having a strategy for emergency response and respiratory support, and a thorough supportive care plan for managing AEs, ensures the team is prepared and confident to approach high acuity treatment. Educating and involving the patient and family when creating a patient‐specific care plan can help to prepare and reduce distress on the infusion day and ensure the patient's needs are fully looked after.

As discussed, administration of premedication and availability of bedside medication, close monitoring of the patient during the infusion, and early recognition of AEs with timely intervention optimizes patient care. Having a well thought out plan in place improves the experience for providers and, more importantly, for the patient and their family.

Finally, it is important to be aware of how much physical space may be required to allow a caregiver/family member and all team members to be close to the patient, all the while ensuring patient comfort and access to all supplies that may be needed during the infusion.

## 4 CONCLUDING REMARKS

The experience of the Levine Children's Hospital team demonstrates that naxitamab can be safely administered in the outpatient setting, and that AEs are manageable when the team is prepared and has the appropriate resources available.

Ensuring that the multidisciplinary team is invested, that the patient and caregivers are involved, and that active communication takes place before, during, and after a patient is treated with naxitamab will help create a positive experience for all involved.

We hope that sharing our clinical experience at Levine Children's Hospital provides practical guidance for institutions looking to treat patients with HR neuroblastoma using naxitamab. While further refinements are anticipated as our team grows in experience, we hope this overview will give care providers the confidence to offer this valuable treatment, knowing that if you plan well, you can be successful.

## AUTHOR CONTRIBUTIONS

**Erin Murphy Trovillion:** Conceptualization (equal); formal analysis (equal); investigation (equal); methodology (equal); supervision (lead); writing – original draft (equal); writing – review and editing (equal). **Giselle L. Saulnier Sholler:** Conceptualization (equal); writing – review and editing (equal). **Javier Oesterheld:** Conceptualization (equal); investigation (equal); writing – review and editing (equal). **Cathryn C. Jordan:** Methodology (equal); project administration (equal); resources (equal); supervision (equal); writing – review and editing (equal). **Meghan Michael:** Methodology (equal); supervision (equal); writing – review and editing (equal). **Katlin Phillips:** Data curation (equal); formal analysis (equal); investigation (equal); project administration (equal); writing – review and editing (equal). **Lauren Brown:** Data curation (equal); formal analysis (equal); investigation (equal); project administration (equal); writing – review and editing (equal).

## CONFLICT OF INTEREST STATEMENT

Erin Trovillion has served on an advisory board for Y‐mAbs Therapeutics, Inc. Meghan Michael has no disclosures. Cathryn Jordan has no disclosures. Lauren Brown has no disclosures. Katlin Phillips has no disclosures. Javier Oesterheld has served on an advisory board for Y‐mAbs Therapeutics, Inc., and Servier Pharmaceuticals. Giselle Saulnier‐Sholler has served as a speaker for Y‐mAbs Therapeutics, Inc.

## Supporting information


Appendix S1.

## Data Availability

N/A.
