# Supplementary material for: Guidelines for outpatient administration of naxitamab: Experience from Atrium Health Levine Children's Hospital
Source: Cancer Med. 2024 Feb 23;13(3):e7045. doi: 10.1002/cam4.7045 (PMC10891358; doi:10.1002/cam4.7045)
Supplement: Supplementary file 1 — Appendix S1. [file CAM4-13-e7045-s001.docx]

# **SUPPLEMENTAL MATERIALS**

## **Appendix 1: Roles and Responsibilities for Naxitamab treatment at Atrium Health Levine Children’s Hospital**


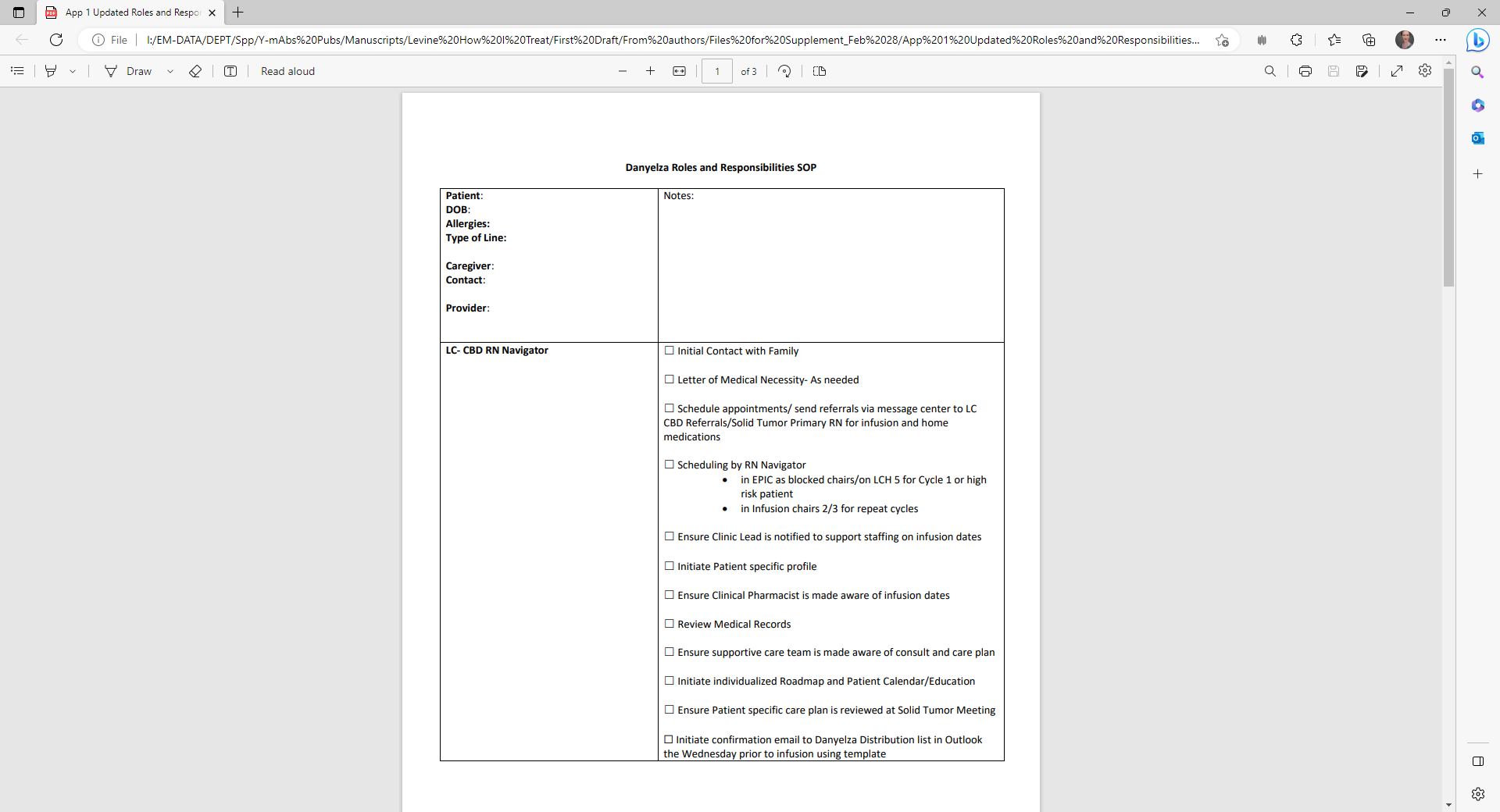


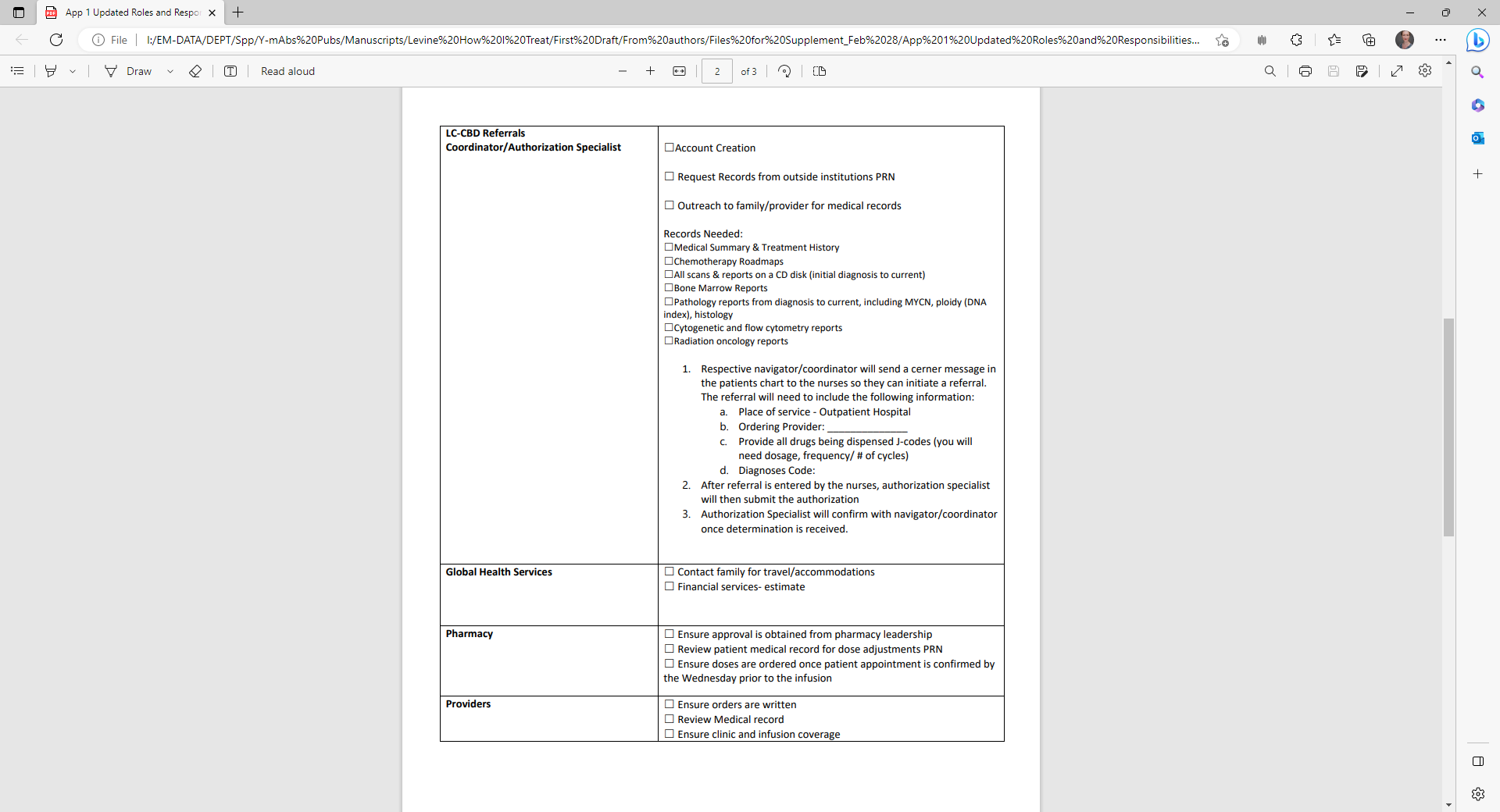


DOB, date of birth; LC-CBD, Levine Children's Cancer and Blood Disorders; PRN, as needed; RN, registered nurse; SOP, standard operating procedure.

## **Appendix 2. New Therapy Submission Form**


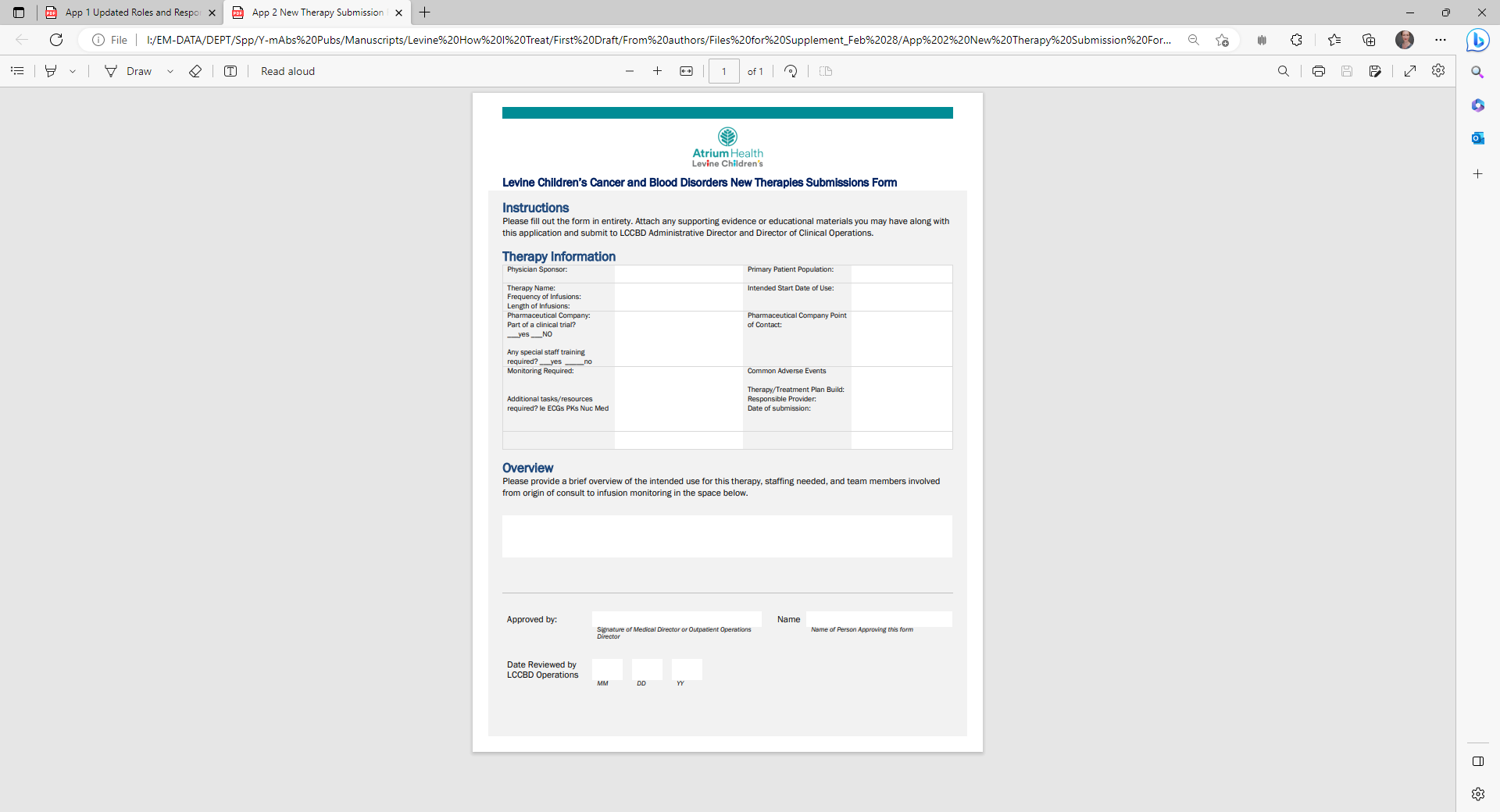
ECG, electrocardiogram; LCCBD, Levine Children's Cancer and Blood Disorders; PK, pharmacokinetics; Nuc Med, Nuclear Medicine.

## **Appendix 3. New Project Timeline**


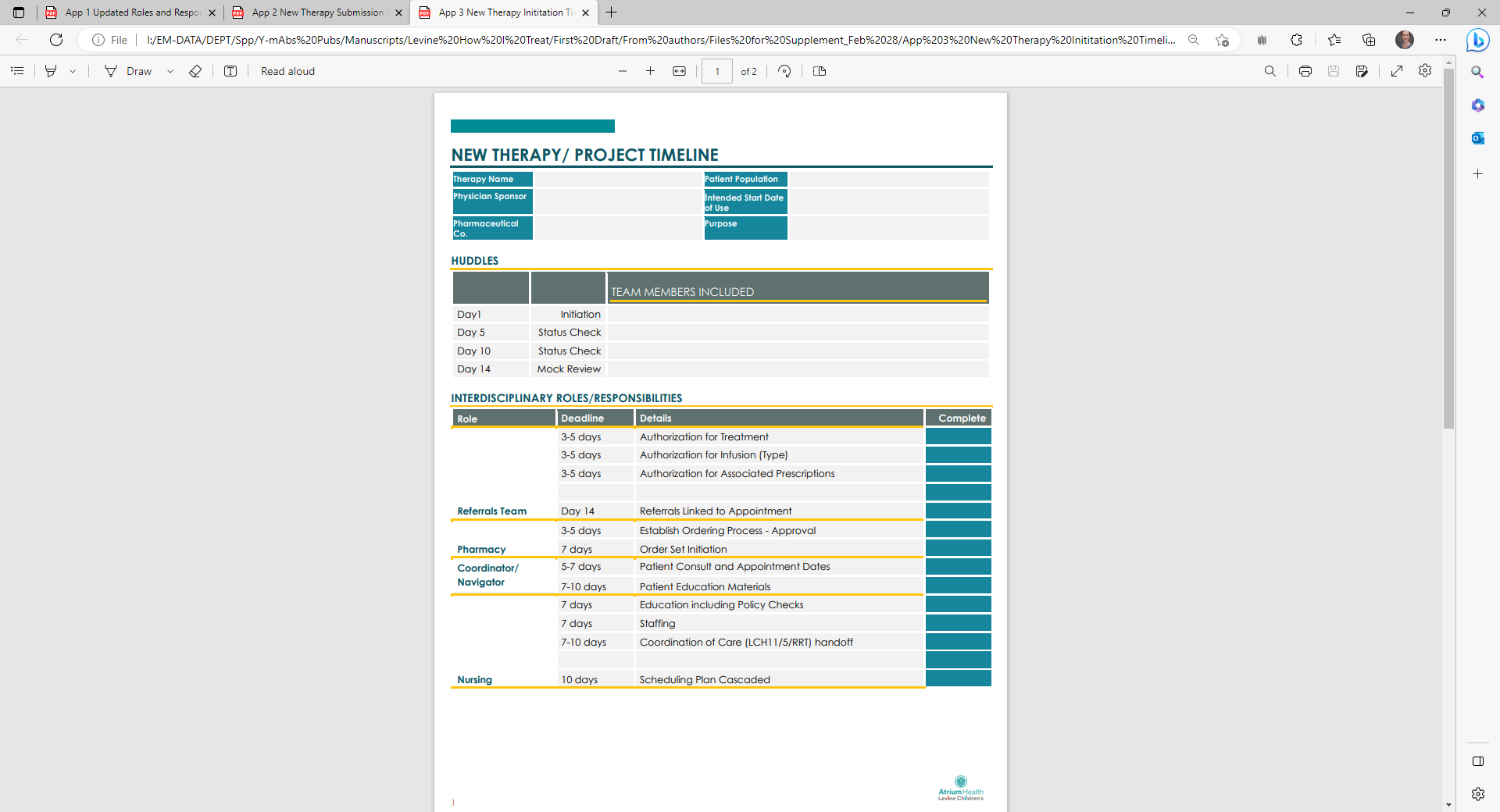


LCH, Levine Children’s Hospital; RRT, registered respiratory therapist.

## **Appendix 4: Protocol: Naxitamab for R/R HR neuroblastoma at Atrium Health Levine Children’s Hospital**


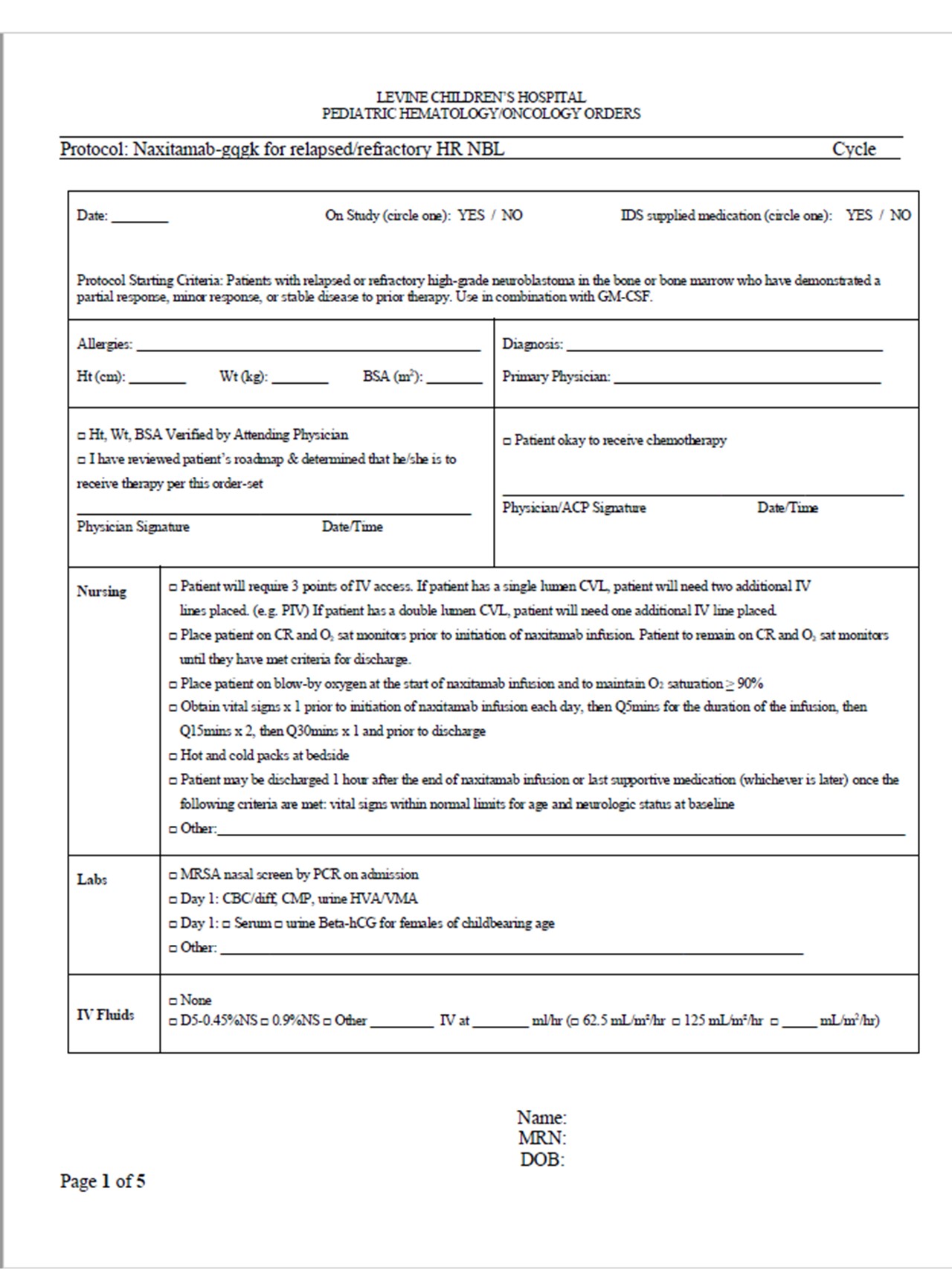


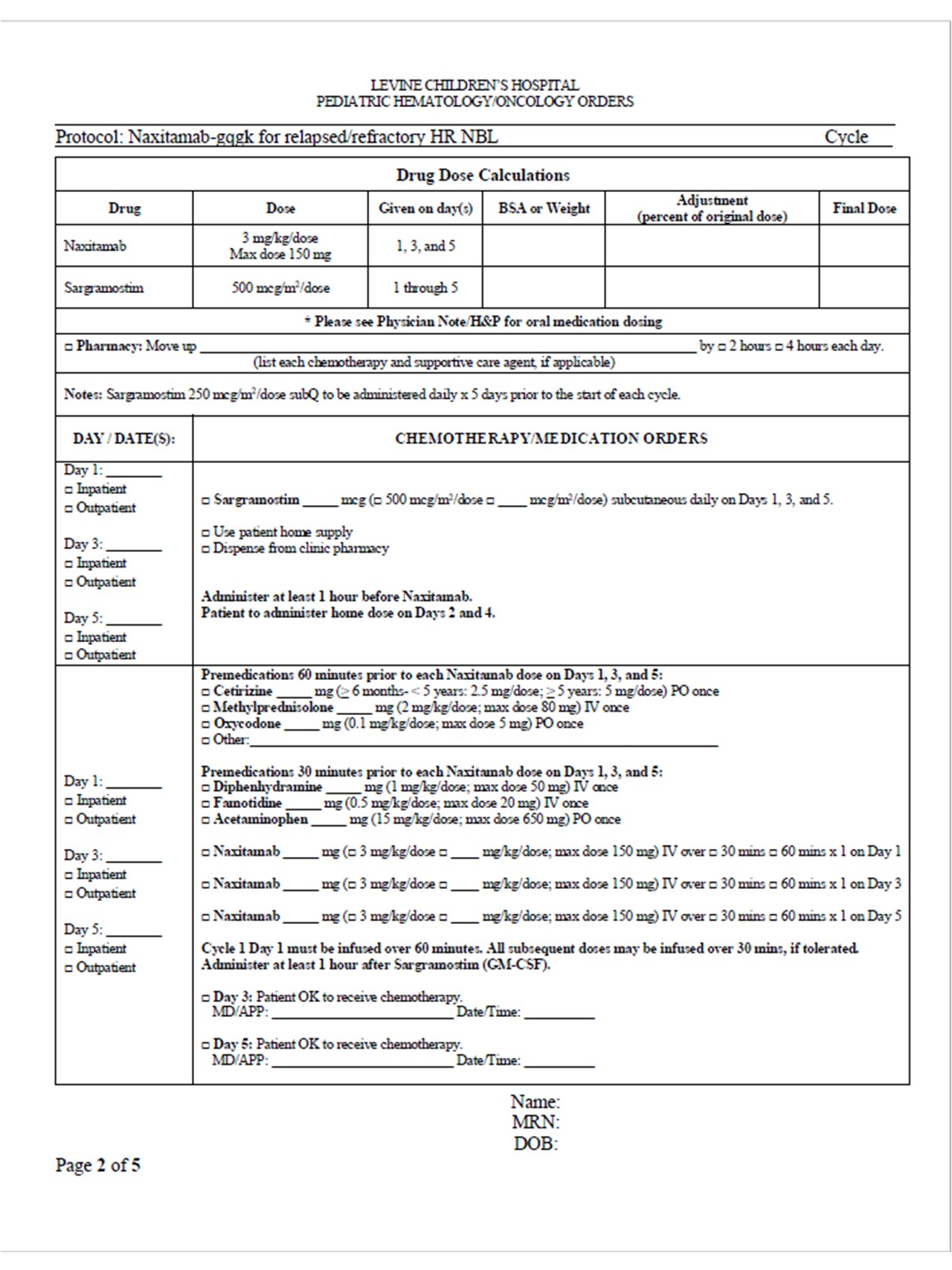

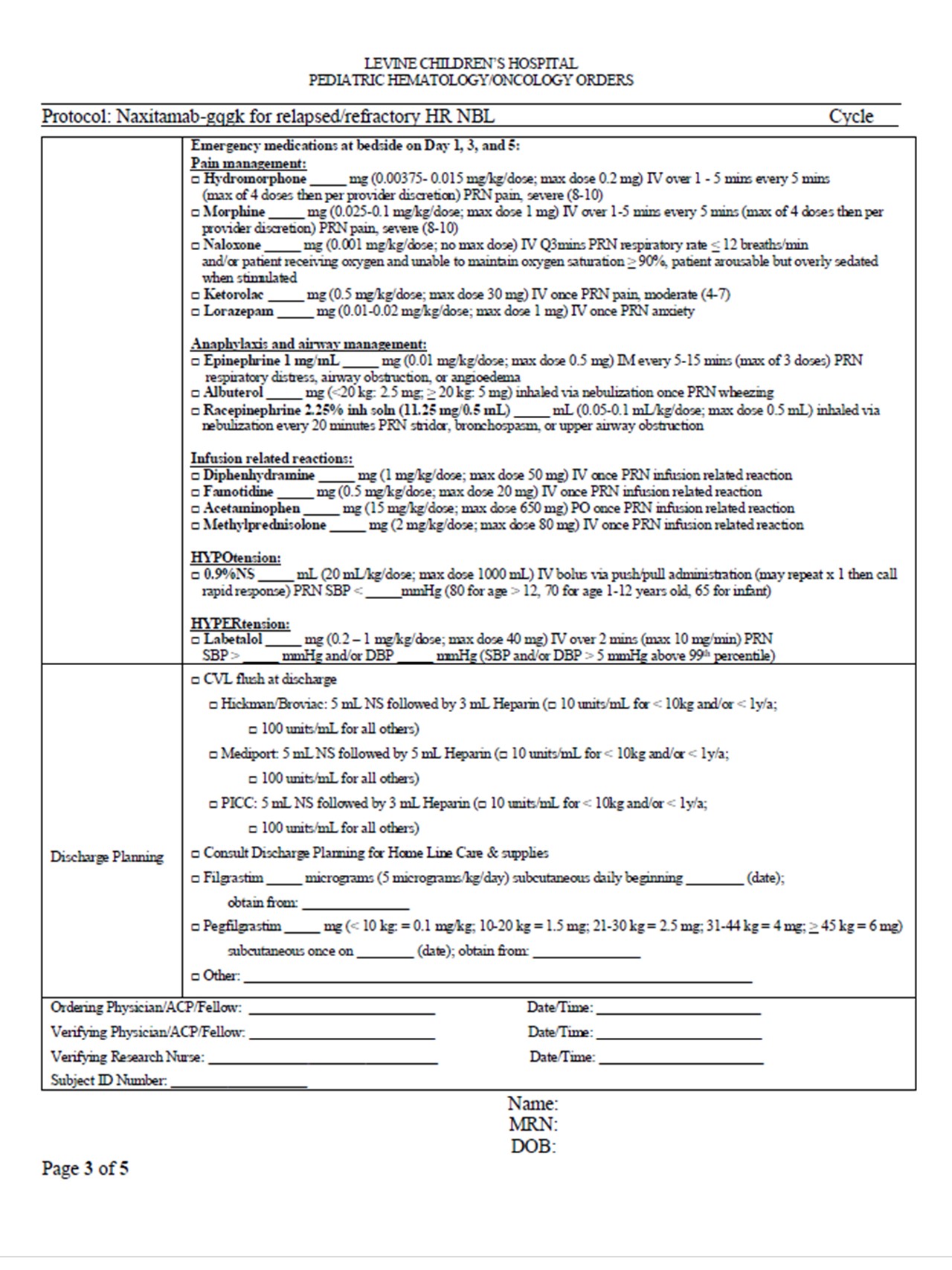


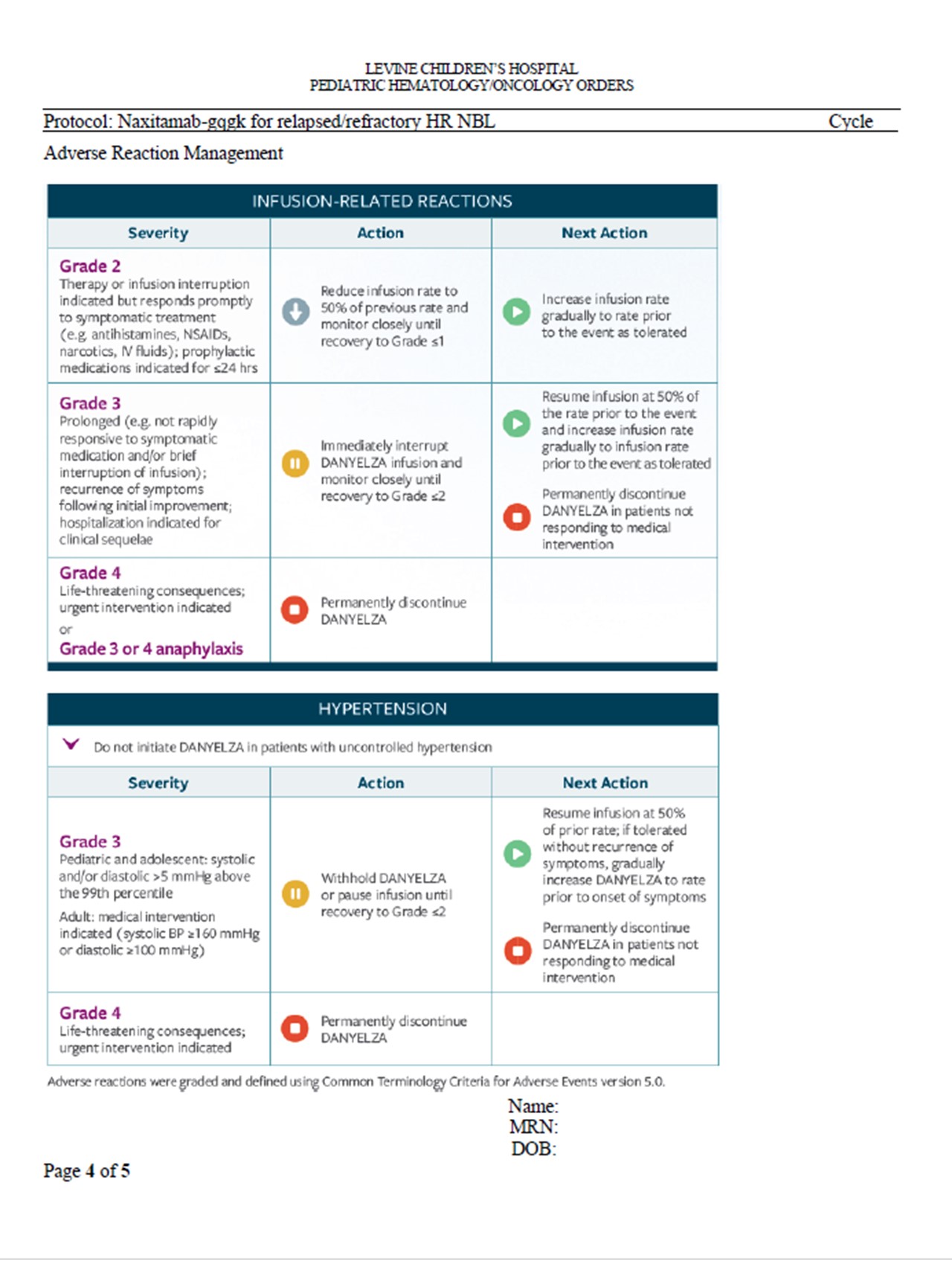


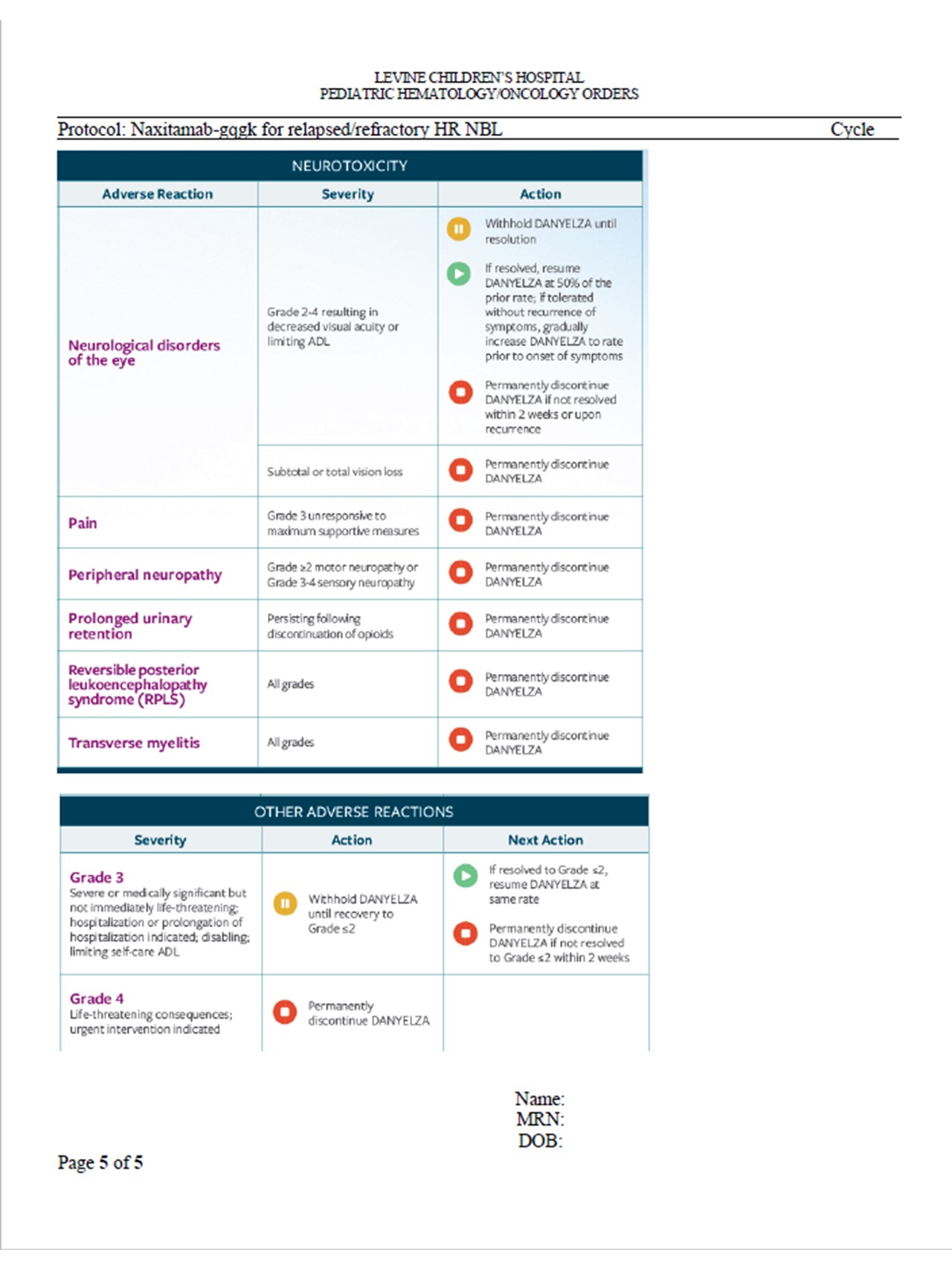


ACP, advanced care provider; ADL, activities of daily living; BP, blood pressure; BSA, body surface area; CBC/diff, complete blood count with differential; CMP, comprehensive metabolic panel; CR, cardiorespiratory; CVL, central venous line; DOB, date of birth; GM-CSF, granulocyte–macrophage colony-stimulating factor; hCG, human chorionic gonadotrophin; H&P, history & physical exam; hr, hour; Ht, height; HR NBL, high-risk neuroblastoma; HVA/VMA, homovanillic acid/vanillylmandelic acid; IDS, Investigational Drug Services; IV, intravenous; MD/APP, medical doctor/advanced practice provider; MRN, medical record number; MRSA, methicillin-resistant Staphylococcus aureus; NA, normal saline; NSAID, nonsteroidal anti-inflammatory drug; PCR, polymerase chain reaction; PICC, peripherally inserted central catheter; PIV, peripheral intravenous; PO, oral administration; Q5/15, every 5/15; PRN, as needed; subQ, subcutaneous; Wt, weight.

## **Appendix 5: Nurse Checklist for Naxitamab Treatment at Atrium Health Levine Children’s Hospital**


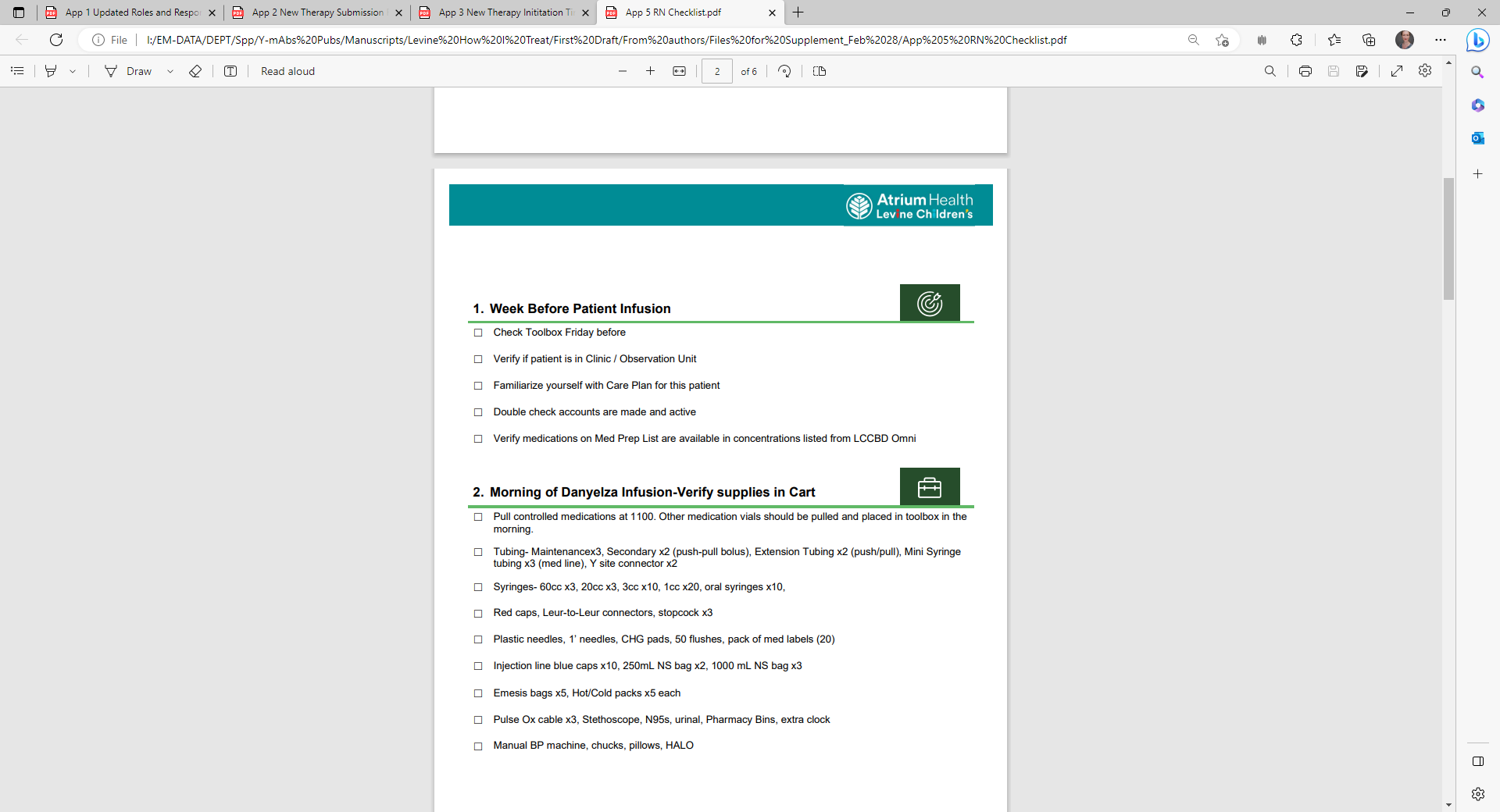


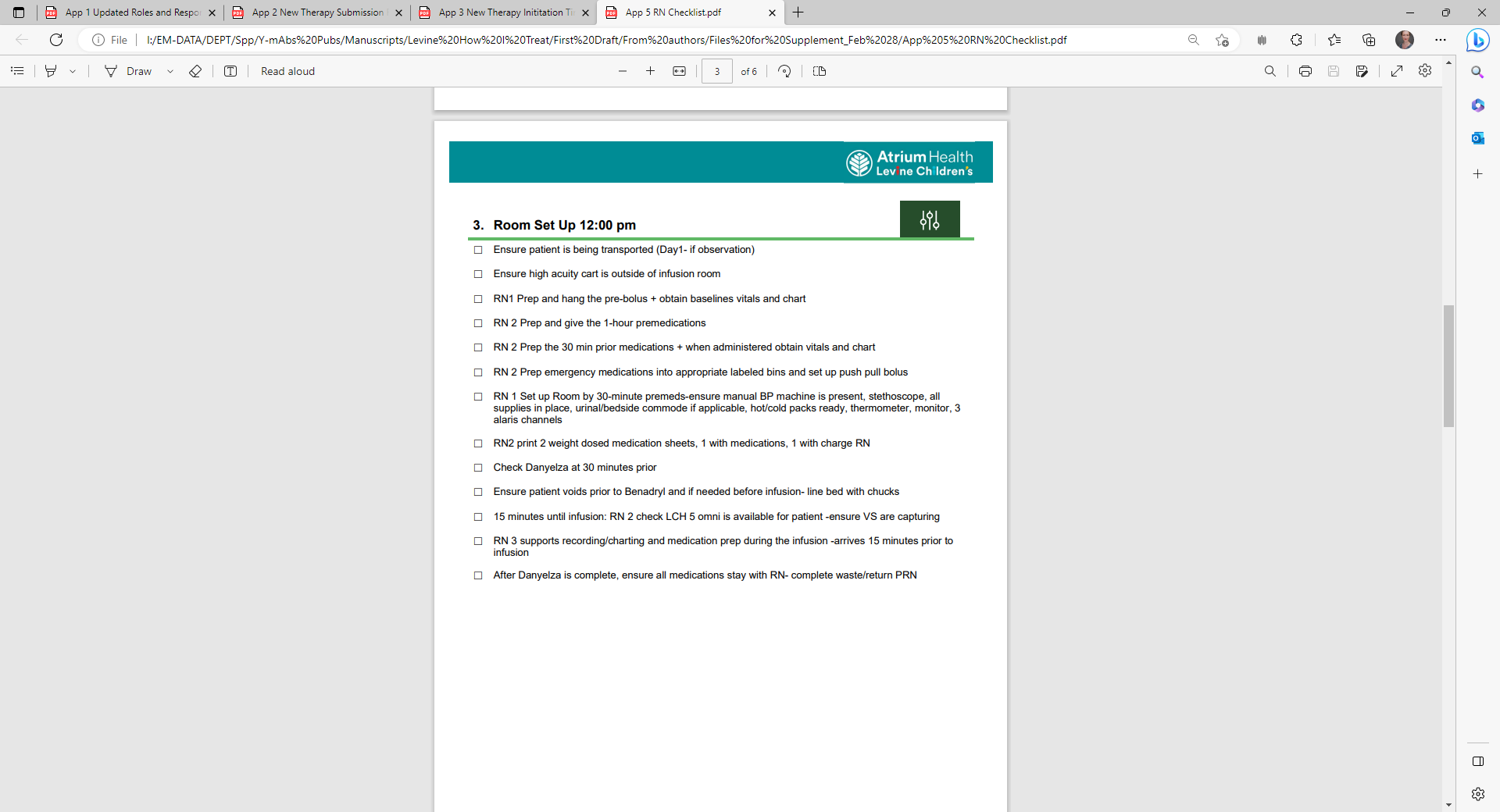


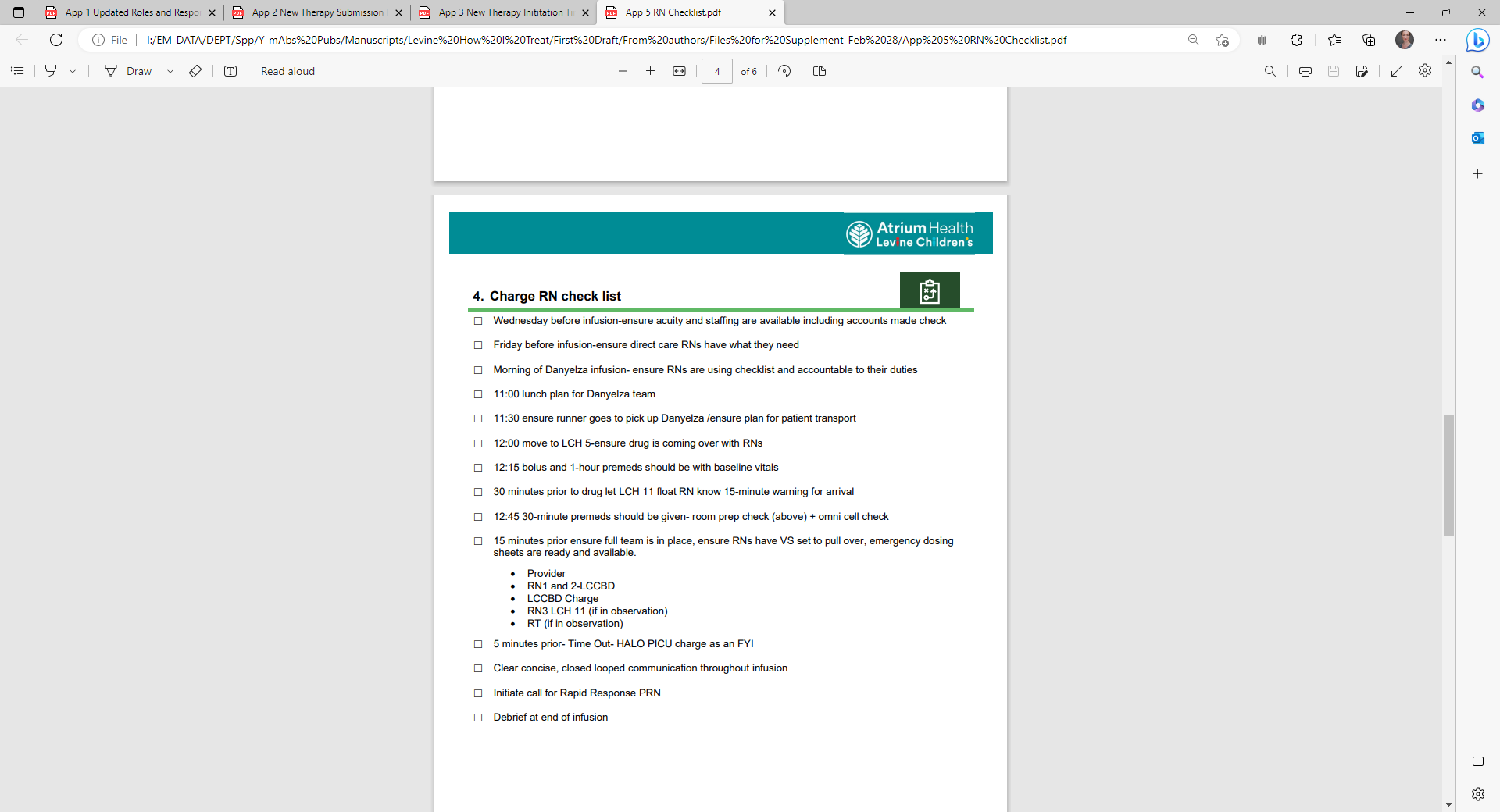


BP, blood pressure; CHG, chlorhexidine gluconate; HALO, secure messaging system used at Atrium Health Levine Children’s Hospital; LCCBD, Levine Children's Cancer and Blood Disorders; LCH, Levine Children’s Hospital; PICU, pediatric intensive care unit; PRN, as needed; RN, registered nurse; VS, vital signs.
